# New Insights into the Fluid Management in Patients with Septic Shock

**DOI:** 10.3390/medicina59061047

**Published:** 2023-05-29

**Authors:** Charalampos D. Moschopoulos, Dimitra Dimopoulou, Anastasia Dimopoulou, Konstantina Dimopoulou, Konstantinos Protopapas, Nikolaos Zavras, Sotirios Tsiodras, Anastasia Kotanidou, Paraskevi C. Fragkou

**Affiliations:** 1 Fourth Department of Internal Medicine, School of Medicine, Attikon University Hospital, National and Kapodistrian University of Athens, 12462 Athens, Greece; bmosxop@yahoo.gr (C.D.M.); sotirios.tsiodras@gmail.com (S.T.); 2 Second Department of Pediatrics, “Aghia Sophia” Children’s Hospital, 11527 Athens, Greece; dimi_med@hotmail.com; 3 First Department of Pediatric Surgery, “Aghia Sophia” Children’s Hospital, 11527 Athens, Greece; 4 Department of Gastroenterology, Hippokration General Hospital, 11527 Athens, Greece; 5 Department of Pediatric Surgery, School of Medicine, Attikon University Hospital, National and Kapodistrian University of Athens, 12462 Athens, Greece; nzavras@med.uoa.gr; 6 First Department of Critical Care Medicine and Pulmonary Services, School of Medicine, Evangelismos Hospital, National and Kapodistrian University of Athens, 10676 Athens, Greece

**Keywords:** fluids, resuscitation, sepsis, septic shock, balanced crystalloids, saline, liberal fluids, restricted fluids

## Abstract

The importance of fluid resuscitation therapy during the early stages of sepsis management is a well-established principle. Current Surviving Sepsis Campaign (SSC) guidelines recommend the early administration of intravenous crystalloid fluids for sepsis-related hypotension or hyperlactatemia due to tissue hypoperfusion, within the first 3 h of resuscitation and suggest using balanced solutions (BSs) instead of normal saline (NS) for the management of patients with sepsis or septic shock. Studies comparing BS versus NS administration in septic patients have demonstrated that BSs are associated with better outcomes including decreased mortality. After initial resuscitation, fluid administration has to be judicious in order to avoid fluid overload, which has been associated with increased mortality, prolonged mechanical ventilation, and worsening of acute kidney injury. The “one size fits all” approach may be “convenient” but it should be avoided. Personalized fluid management, based on patient-specific hemodynamic indices, provides the foundations for better patient outcomes in the future. Although there is a consensus on the need for adequate fluid therapy in sepsis, the type, the amount of administered fluids, and the ideal fluid resuscitation strategy remain elusive. Well-designed large randomized controlled trials are certainly needed to compare fluid choices specifically in the septic patient, as there is currently limited evidence of low quality. This review aims to summarize the physiologic principles and current scientific evidence regarding fluid management in patients with sepsis, as well as to provide a comprehensive overview of the latest data on the optimal fluid administration strategy in sepsis.

## 1. Introduction

Sepsis is defined as a life-threatening organ dysfunction caused by a dysregulated host response to infection, whereas septic shock is a subset of sepsis in which profound circulatory, cellular, and metabolic abnormalities are associated with a higher risk of mortality than with sepsis alone [1]. Although the exact worldwide burden of sepsis is difficult to ascertain, it certainly represents a major global health issue. In 2017, there was an estimate of 48.9 million cases of sepsis; during the same year, 11 million sepsis-related deaths were reported worldwide, representing almost 20% of global deaths [2]. Between 1990 and 2017, age-standardized sepsis incidence fell by 37% and mortality decreased by 52.8% [2]. Despite these trends, sepsis still remains a major cause of death worldwide. Interestingly, there are significant regional disparities in sepsis-related incidence and mortality, with approximately 85% of sepsis cases and sepsis-related deaths occurring in low- and middle-income countries [2].

The management of sepsis has not significantly changed over the past 40 years. Current guidelines recommend the early administration of antibiotics and intravenous (IV) fluids, in addition to source control and the judicious use of vasopressors [3]. Fluid resuscitation therapy represents one of the cornerstones of sepsis management [3]. Understanding the pathophysiology of sepsis is crucial in order to determine the role of intensive fluid administration in the initial phase of septic shock.

Although there is a consensus on the need for adequate fluid therapy in sepsis and despite the multiple recent clinical trials examining fluid management in sepsis, the ideal fluid management strategy is still controversial and elusive, as there are no clear guidelines about the optimal fluid resuscitation in critically ill patients with sepsis. The purpose of this narrative review is to summarize the physiologic principles and current scientific evidence regarding fluid management in patients with sepsis, as well as to provide a comprehensive overview of the latest data on the optimal type (balanced crystalloids versus normal saline) and volume (liberal versus restricted administration) of fluids in sepsis and septic shock patients.

## 2. Methods

A literature search was performed to identify all published research, such as original articles, reviews, and systematic reviews/metanalyses, using the key words “fluid resuscitation”, “sepsis”, “septic shock”, “critically-ill”, balanced crystalloids”, “normal saline”, “liberal fluid administration”, and “restricted fluid administration”. Records were retrieved from PubMed/Medline and Scopus, without prior application of language or other restrictions for the database search. Reference lists of included articles were also screened to identify potential studies missed by the initial literature search.The physiology of fluid administration in sepsis is a complex syndrome with multiple underlying mechanisms contributing to its pathogenesis. The initial step involves the recognition and the binding of pathogen-associated molecular patterns (PAMPs) by pattern recognition receptors (PRRs) on the surface of host immune cells [4,5]. Toll-like receptors (TLRs) are a crucial group of PRRs that play a significant role in initiating the immune response [4,5]. They recognize various PAMPs such as bacterial lipopolysaccharide, viral RNA, and fungal cell wall components [4,5]. PRRs can also recognize endogenous danger-associated molecular patterns (DAMPs) that are released during the inflammatory insult [4,5]. Upon pathogen recognition, immune cells release pro-inflammatory cytokines, such as tumor necrosis factor alpha (TNF-α), interleukin-1 beta (IL-1β), and interleukin-6 (IL-6) [4,6]. These cytokines trigger a cascade of immune responses and recruit additional immune cells to the site of infection, such as polymorphonuclear leukocytes and macrophages [4,6]. In sepsis, the immune response becomes dysregulated, leading to an excessive and uncontrolled release of pro-inflammatory cytokines, commonly referred to as a cytokine storm. This hyperinflammatory state contributes to tissue damage and organ dysfunction [4,6]. Simultaneously, there is an activation of the anti-inflammatory response, resulting in immunosuppression by inhibiting cytokine production by mononuclear cells and monocyte-dependent T helper cells [4,6].

Sepsis is characterized by a dysregulated inflammatory response with derangement of both macro- and microcirculation, leading to a status of actual and relative hypovolemia, resulting in tissue hypoperfusion and imbalance between oxygen delivery and demand [7,8]. The absolute and relative intravascular volume depletion in septic patients is attributed to gastrointestinal fluid losses, insensible loss from tachypnea, anorexia with decreased oral intake, arterial vasodilation, cytokine-mediated injury of the endothelium leading to capillary leaks and fluid extravasation to the interstitial compartment, and venodilation with increased venous capacitance [9,10,11]. All these mechanisms can be present in varying degrees among patients and are responsible for the reduction in stressed volume, venous return, and therefore, ventricular preload and cardiac output, which further promotes tissue hypoxia [11,12].

Fluid administration is considered the cornerstone of initial hemodynamic resuscitation in sepsis in order to restore circulating fluid volume and increase cardiac output and eventually, oxygen delivery [13,14]. Fluid resuscitation exerts its therapeutic effect if the augmentation of stressed volume results in a pressure gradient for venous return that exceeds the central venous pressure (CVP) [14]. The subsequent increase in cardiac output restores the mean arterial pressure (MAP) and thus, improves microcirculatory flow and perfusion pressure, reducing the risk of tissue hypoperfusion and subsequent ischemic damage [15]. This concept is consistent with the Frank–Starling principle which demonstrates that under normal physiological circumstances, increasing the preload could optimize stroke volume, even though under a septic state, this therapeutic benefit may be affected [11,16]. The restoration of intravascular volume supports the renal function, increasing diuresis and the clearance of metabolic waste products [17]. Furthermore, fluid resuscitation contributes to the stabilization of electrolyte and acid–base balance, resulting in the maintenance of cellular homeostasis [17]. Fluid resuscitation also aims to maintain the microvascular integrity and endothelial function, preventing endothelial barrier dysfunction and reducing tissue edema [18]. These effects are essential in promoting oxygen and nutrient delivery to the tissues and supporting organ function during critical illness and sepsis [18].

This reasonable and well-established mechanism is excessively simplistic and it is increasingly recognized that, apart from hemodynamics, oxygen delivery and organ perfusion are also affected by the bioenergetic failure and impaired oxidative metabolism [19,20]. This is best demonstrated in the septic heart, where aggressive volume resuscitation may paradoxically result in cardiovascular collapse and impaired myocardial contractility. This can be explained by mitochondrial oxidative stress, microvascular thrombosis, and increased myocardial edema [21,22,23]. Indeed, despite an apparent initial improvement after fluid administration, eventually, half of septic patients become non-responsive to fluids, experiencing significant harmful effects such as fluid extravasation, decreased venous return, and impaired tissue perfusion, with a minimal increase in end-diastolic volume [24,25]. In addition, while the purpose of fluid resuscitation is the improvement of hemodynamic parameters with CVP > 8 mmHg and MAP > 65 mmHg, a compensatory decrease in systemic vascular resistance (SVR) may occur, worsening the clinical outcomes [19,26]. This has been attributed to the inhibition of sympathetic activity and the augmentation of endothelial shear stress in response to fluid administration, which are followed by an increase in nitric oxide release and, ultimately, vasodilation [27,28,29]. Fluid bolus administration may result in a decrease in SVR through hemodilution and reduced blood viscosity [15].

Excessive fluid resuscitation may exacerbate shock, as a significant increase in filling pressures can overcome cardiac compensation mechanisms when the patient reaches the plateau of the Frank–Starling curve [19,30,31]. The subsequent high left atrial pressure leads to pulmonary congestion and edema, the potential development of pulmonary hypertension, and finally, left ventricle dysfunction and a reduction in left-ventricular volume and cardiac output [32,33]. Similarly, high right atrial pressure results in a decrease in venous return and retrograde increased venous pressure. This results in fluid extravasation into the interstitial space and subsequent tissue edema, which leads to architecture distortion and increased resistance to capillary blood flow and lymphatic drainage [13,14,28].

Moreover, sepsis produces alterations in vascular permeability and only 5% of fluid bolus volume remains intravascular after 90 min in the critically ill patient, due to rapid fluid redistribution [30]. It is well established that sepsis leads to damage of the endothelial glycocalyx, which is a main determinant of membrane permeability and vascular homeostasis [13,34,35]. Glycocalyx degradation may be exacerbated due to fluid overload, as the increase in cardiac filling pressures results in the release of natriuretic peptides that cleave proteoglycans and glycoproteins from the glycocalyx [36,37]. Consequently, the high capillary leak translates clinically into inadequate and short-lived responses of hemodynamic parameters to fluid administration with increased tissue edema, decreased oxygen diffusion, and possible organ failure [14,38,39]. The better understanding of the complexity of septic patient response to fluid resuscitation, especially after the recent recognition of the role of endothelial glycocalyx, has led to the development of a revised Starling principle [9,20]. This new model demonstrates the important role of glycocalyx for transvascular fluid exchange, allowing for more efficient therapeutic strategies to be designed in order to improve patient outcomes [40].

The volume and the type of fluid used for initial resuscitation and the maintenance of fluid therapy have an impact on salt and water retention and by extension to fluid overload, which affects all the major organ systems, causing potential unfavorable outcomes on end-organ function [41,42]. More specifically, aggressive fluid resuscitation may result in secondary intra-abdominal hypertension, which is associated with acute kidney injury, hepatic venous congestion, respiratory dysfunction with pulmonary edema, as well as disorders in the central nervous system, such as cerebral edema and intracranial hypertension and in the cardiovascular system, such as myocardial edema, decreased ejection fraction, and cardiac output, leading to multi-organ failure and death [19,43,44].

## 3. Fluid Resuscitation in Sepsis

Despite the scientific advances of the last 20 years, sepsis management has not changed drastically, apart from the introduction of the bundles, which designate multiple interventions that should be completed within a specific time frame. After initial airway and respiratory stabilization, sepsis bundle should be performed within the first 3 h of presentation. The SSC 2021 bundle includes fluid resuscitation, antibiotic administration, lactate measurement and obtainment of cultures [3]. Vasopressors should be initiated if the patient remains hypotensive despite adequate fluid resuscitation [3]. However, a group of 34 European Society of Intensive Care Medicine (ESICM) experts recently suggested to start vasopressors early, before full completion of fluid resuscitation [45]. In the revision of the Surviving Sepsis Campaign (SSC) guidelines in 2018, the 3 and 6 h bundles were combined into a single “1-h bundle” where fluid resuscitation is required in all patients without exception [46]. The implementation of these sepsis protocols in clinical practice have led to decreased sepsis mortality [47].

Fluid resuscitation remains an integral part of sepsis management, since it was first employed during the European cholera epidemic as early as 1830 [48]. The following years, fluid resuscitation was used to treat hypovolemia and restore tissue perfusion pressure in order to improve oxygen transport to cells [49]. Previous versions of SSC guidelines recommended a quantitative resuscitation protocol, that was based entirely on the early goal-directed therapy (EGDT) study [50]. This landmark study showed the benefit of early and aggressive fluid resuscitation in the mortality and the maintenance of a CVP of 8–12 mmHg and a central venous oxygen saturation (S_CV_O2) of at least 70% [50]. The era of a time-sensitive bundled care was then introduced in sepsis. However, subsequent multi-center randomized controlled trials (RCTs) failed to reproduce the benefits observed in the EGDT trial [51].

**Figure 1:**
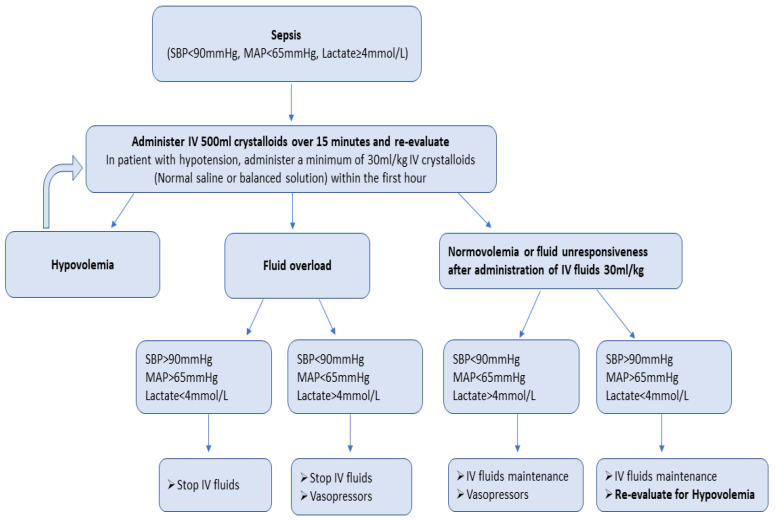
Proposed algorithm of fluid resuscitation in patients with sepsis.

**Figure 2:**
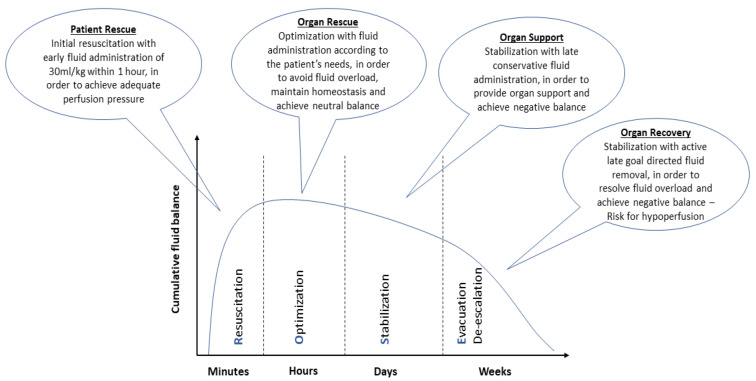
Characteristics of the four distinct phases of intravenous fluid therapy: resuscitation, optimization, stabilization, and evacuation (ROSE).

There is a growing scepticism regarding aggressive fluid resuscitation, since this approach may lead to massive fluid overload and, inevitably, to adverse outcomes [52]. An increasing number of studies have associated fluid overload to worse outcomes and increased mortality in septic patients [31,33,53]. Current SSC guidelines recommend the early administration of 30 mL/kg of IV fluids for sepsis-related hypotension or a lactate ≥ 4 mmol/L, within the first 3 h of resuscitation [3]. This recommendation remains weak, as it is based on low-quality evidence. Infusing an initial 1 L bolus over the first 30 min and administrating the remainder volume of fluid resuscitation with repeated bolus infusions is an acceptable approach [54]. A proposed algorithm about fluid resuscitation in patients with sepsis is shown in Figure 1 [3,8,10]. Four distinct phases of IV fluid therapy have been proposed: resuscitation, optimization, stabilization, and evacuation (ROSE), which are all crucial steps in sepsis management (Figure 2) [55]. In addition, specific strategies for fluid minimization and de-escalation or de-resuscitation have been reported, demonstrating that fluid restriction is associated with improved outcomes [30,56].

The 2021 SSC guidelines suggest the use of crystalloid fluids [3]. However, different types of fluids have been proposed. Colloids, including albumin and semisynthetic colloids, such as hydroxyethyl starch (HES), dextrans, and gelatins, were commonly used in the past. Several studies which examined their use in septic patients recommend against the administration of HES and other semisynthetic colloids [57,58,59,60,61]. HES use has been associated with acute kidney injury and the need for renal replacement therapy, as well as with increased mortality [61]. Gelatins have been found to increase anaphylaxis, renal failure, bleeding, and mortality [62]. Hence, the side effects of semisynthetic colloids far outweigh any potential benefits and, according to the SSC guidelines, their use should be avoided in sepsis management [3].

Current SSC guidelines suggest using albumin in septic patients who received large volumes of crystalloids over using crystalloids alone [3]. Albumin is not recommended as the first-line fluid for resuscitation in sepsis due to the lack of proven benefit and its higher cost compared to crystalloids [3]. However, two RCTs, the Saline versus Albumin Fluid Evaluation (SAFE) and the Albumin Italian Outcome Sepsis (ALBIOS) study, as well as a meta-analysis of randomized clinical trials, compared the effect of albumin and crystalloid use in patients with sepsis or septic shock, and showed a trend towards reduced mortality and improved outcomes in the albumin group, without observing serious side effects [63,64,65].

In septic patients, human albumin solution can be given for two indications: to restore or expand intravascular volume and to supplement serum albumin in the septic patients with hypoalbuminemia [66]. In addition, human albumin acts as the most significant modulator of plasma oncotic pressure, which is typically in the 25–30 mmHg range. This is a major endogenous antioxidant agent and a major binding protein of several endogenous compounds and drugs [66]. Albumin appears to have important immunomodulatory effects that likely impact the host inflammatory response in critical illness [66]. The time, dose, and concentration of the albumin, as well as the determination of a specific target for serum albumin level remains controversial. Of note, in the ALBIOS trial, albumin was administered as a 20% solution, with a treatment goal of a serum albumin concentration of 30 g/L until intensive care unit (ICU) discharge or 28 days [64].

## 4. Balanced Crystalloids versus Normal Saline in Sepsis and Septic Shock

The ideal fluid for septic patients, which would have similar osmolarity to plasma, increase intravascular volume and cardiac output, and improve tissue perfusion without causing tissue edema, while at the same time be cost effective, has yet to be discovered [9].

Fluids are classified according to their composition in two major categories: crystalloid and colloid solutions. Crystalloids are recommended as first-line resuscitation fluids in patients with sepsis as they are inexpensive, widely available, and lead to fewer serious adverse effects [67,68]. Isotonic crystalloids have a tonicity similar to plasma and are further divided into balanced and unbalanced solutions. Herein, we summarize the current evidence regarding the use of the two different types of crystalloids in patients with sepsis and septic shock, and their effects on patient outcomes (Table 1).

### 4.1. Unbalanced Solutions

The most commonly used unbalanced solution is 0.9% normal saline (NS). It is an isotonic solution and contains equal concentrations of sodium and chloride (154 mmol/L) and no organic anion to act as acid buffer. As a result, it has a strong ion difference equal to zero. Notably, its chloride concentration is almost 40% higher than that of plasma. This high chloride concentration has been associated with hyperchloremic metabolic acidosis, impaired tissue and renal perfusion, acute kidney injury, coagulopathy, and altered inflammatory response [69,70,71,73,74].

### 4.2. Balanced Solutions

Balanced solutions (BSs), apart from sodium and chloride, include other ions, such as potassium, calcium, and magnesium, and may also contain buffers such as bicarbonate, lactate, acetate, or gluconate, leading to electroneutrality (balance between positive and negative anions) [75]. Moreover, a crystalloid solution is considered balanced when it has a strong ion difference close to 24 mEq/L and contains chloride similar to plasma’s chloride concentration (98–112 mmol/L) [76]. Commonly used BSs are lactated Ringer’s, Hartmann’s solution, Plasma-Lyte, and Normosol. More specifically, lactated Ringer’s solution consists of sodium, chloride, potassium, calcium, and sodium lactate mixed into a solution with an osmolality of 273 mOsm/L and pH of 6.5 [77]. Hartmann’s solution is similar to lactated Ringer’s, while the main difference of Plasma-Lyte is that it does not contain calcium [78]. Finally, Normosol, like Plasma-Lyte, is calcium-free solution and is composed of sodium, potassium, magnesium, chloride, acetate, and gluconate, while its pH is 7.4 [79].

### 4.3. Studies Comparing BSs versus NS in Sepsis and Septic Shock

Numerous studies have been carried out in order to investigate and compare the effectiveness of BSs and NS on septic patients’ outcomes and identify the optimal fluid solution. However, which type of crystalloid solution should be administered during the management of patients with sepsis and septic shock remains unanswered, due to the low quality of existing evidence. Current SSC guidelines strongly recommend crystalloids as first-line fluid resuscitation, and further suggest using a BS instead of NS for the management of patients with sepsis or septic shock (weak recommendation, low quality evidence) [3].

A double-blind, cluster randomized, double-crossover trial was conducted in four ICUs in New Zealand during a 7-month period (the Saline vs. Plasma-Lyte 148 for ICU fluid Therapy (SPLIT) trial) aiming to determine the effect of BS in comparison with NS on acute kidney injury [80]. The results of this study did not reveal significant differences in the outcomes of incidence of acute kidney injury or mortality among critically ill patients who received BS or NS; however, among the enrolled patients, only 4% were septic [80]. On the other hand, another cluster-randomized, multiple cross-over trial (the isotonic Solution Administration Logistical Testing (SALT) trial) compared the impact of BSs versus NS on patient outcomes in ICU and showed that patients who received larger volumes of NS appeared to experience more frequent major renal complications; the proportion of septic patients who received a BS and NS was 28.6% and 25%, respectively [81].

In 2018, the single-center cluster-randomized isotonic Solutions and Major Adverse Renal Events Trial (SMART) was conducted in five ICUs and compared the effect of BS and NS on mortality and renal outcomes [82]. This was the first RCT, demonstrating that the IV administration of BS during fluid resuscitation was associated with lower mortality and more favorable outcomes regarding the need for renal replacement therapy and persistent renal dysfunction, compared to NS [82]. More specifically, a secondary analysis of the SMART trial which focused on critically ill adults with sepsis revealed that the use of BSs was associated with a lower 30-day in-hospital mortality compared to NS [83]. Additionally, an ancillary analysis of the SMART trial demonstrated that the use of BSs was associated with a modest decline in early biomarkers of acute kidney injury [72].

Conversely, the large RCT Balanced Solution in Intensive Care Study (BaSICS), which aimed to determine the effect of BS (Plasma-Lyte 148) or NS on 90-day survival in ICU patients, did not reveal any significant reduction in mortality between the two groups [84]. Interestingly, a secondary post hoc analysis demonstrated that, especially in the subgroup of septic patients who exclusively received BS before trial enrolment, the probability of 90-days survival was higher. As a result, the type of fluid used for the initial resuscitation may alter the outcomes in septic shock patients, as these patients seem to be more sensitive to external chloride overload possibly due to the decreased albumin synthesis caused by the inflammatory response [85].

Recently, another RCT, the Plasma-Lyte 148 versus Saline Study (PLUS) trial examined the relationship between the administration multi-electrolyte BS (Plasma-Lyte 148) versus NS, and the outcomes of 90-day mortality and renal complications in critically ill adults [86]. Notably, 42.3% of the enrolled patients had sepsis. In contrast to other trials, the PLUS trial did not find any significant difference between the two types of fluids [86].

Multiple observational, retrospective, and cohort studies have also been published, comparing the use of BS and NS. Specifically, a retrospective cohort study was designed to determine the effect of a BS (Normosol) compared with NS on the outcomes of patients with sepsis, defined as acute kidney injury and the need for renal replacement therapy [79]. This study did not reveal any difference among septic patients who were resuscitated with either Normosol or NS [79]. Similarly, no statistically significant difference was observed among septic patients treated with lactated Ringer’s solution or NS regarding serum lactate clearance, serum creatinine change within 24 h, and 48 h survival after admission at the emergency department [87]. On the contrary, other studies support the use of BS over NS in septic shock patients, as NS might be associated with renal complications, increased bleeding risk, and mortality [68,88,89,90].

Various systematic reviews and meta-analyses have been conducted comparing the two fluid treatments. Of note, the vast majority of these studies did not reveal any significant difference in the effect of BS or NS on the outcomes of patients with sepsis [91,92,93,94]. However, the most recent meta-analysis demonstrated that BS administration in septic patients was associated with decreased mortality and acute kidney injury as compared with NS, although subgroup analysis including only RCTs did not show any difference between the two groups [95].

Despite the abundance of data regarding the effectiveness and safety of BS and NS use on adults with sepsis, few studies have been carried out in children. Currently, NS remains the preferred resuscitation fluid for children with sepsis, although the SSC guidelines suggest the use of BS rather than NS for the initial resuscitation (weak recommendation, very low quality of evidence) [96]. Due to the scarcity of evidence to support BS or NS, a large Pragmatic Pediatric trial of Balanced versus NS Fluid in sepsis (PRoMPT BOLUS) is now being conducted in order to establish clear, high-quality evidence and determine whether the use of BS in pediatric patients with sepsis is associated with improved outcomes compared to NS [97].

## 5. Liberal versus Restricted Fluid Administration in Sepsis and Septic Shock

Fluid resuscitation is of paramount importance during the early stages of sepsis and septic shock management in order to address the deficit in effective vascular volume and the resulting tissue hypoperfusion. Several RCTs have been designed to investigate the timing and amount of fluid administration, as well as to identify appropriate target goals to guide fluid resuscitation (Table 2). Since the ground-breaking EGDT trial by Rivers et al. [50], this has become common practice, and the SSC guidelines currently suggest that at least 30 mL/kg (ideal body weight) of IV crystalloids should be administered within the first 3 h of sepsis-induced hypoperfusion or septic shock [3]. After initial resuscitation, however, fluid administration has to be balanced in order to avoid positive fluid balance and fluid overload, which has been associated with increased mortality, prolonged mechanical ventilation (MV), and worsening of acute kidney injury [98,99,100]. Furthermore, the EGDT approach itself has been under scrutiny, since three RCTs, namely the Australasian Resuscitation in Sepsis Evaluation (ARISE) [101], the Protocolized Care for Early Septic Shock (ProCESS) [102], and the Protocolised Management in Sepsis (ProMISe) [103] trial, showed that the goal of S_CV_O_2_ > 70% did not improve mortality, but instead resulted in a worse sequential organ failure assessment (SOFA) score, more days on cardiovascular support, and a longer ICU stay. However, in these trials the pre-randomization administered fluids were close to the 30 mL/kg goal, underscoring the wide adoption of SSC guidelines in the clinical practice [104].

On the other hand, trials conducted in resource-limited settings point towards an excess mortality in patients that received bolus fluid therapy. The Fluid Expansion as Supportive Therapy (FEAST) study, conducted in Africa, found an increased risk of death among children with sepsis who received early treatment with bolus 5% albumin or 0.9% saline, in comparison with the control group [105]. Similarly, the Simplified Severe Sepsis Protocol (SSSP) [106] and the Simplified Severe Sepsis Protocol 2 (SSSP-2) [107] trials, which included African adults with sepsis and hypoperfusion, also showed increased mortality in the intervention arm. The application of an early resuscitation protocol resulted in greater IV fluid administration, vasopressor use, and lactate reduction, but caused worsening hypoxemia and higher mortality, leading to the early termination of the SSSP trial. Of note, the majority of patients in these trials had human immunodeficiency virus (HIV) infection with low CD4^+^ counts, and were admitted in regular medical wards without access to MV, conditions that prevent the generalization of these results in higher resource settings.

Increased attention has recently been drawn to the optimal fluid management after the initial resuscitation. It has been shown that a higher volume of fluid during the first 3 h, but lower volume in the first 24 h, reduces mortality in severe sepsis and septic shock patients, and that positive total fluid balance increases mortality by 1.7 times [108]. In order to avoid the detrimental effects of fluid overload, further fluid administration should be guided by careful assessment of intravascular volume and organ perfusion [3]. A simple, resource-independent way to assess tissue perfusion is by measuring the capillary refill time (CRT), either on the fingertip or the earlobe [109,110]. In the ANDROMEDA-SHOCK trial, CRT improvement has been found to be a better marker for resuscitation guidance in comparison with the decrease in lactate [111]. In this study, CRT-guided resuscitation resulted in a significantly lower SOFA score at 72 h, and in a lower 28-day mortality that did not reach statistical significance. The ongoing ANDROMEDA-SHOCK-2 trial investigates whether CRT-guided resuscitation based on clinical and hemodynamic phenotypes may decrease mortality in early septic shock patients [112]. The Conservative versus Liberal Approach to Fluid Therapy of Septic Shock in Intensive Care (CLASSIC) [113] and the Crystalloid Liberal or Vasopressors Early Resuscitation in Sepsis (CLOVERS) [114] trials were designed to address whether a restrictive versus a liberal approach in fluid management would improve outcomes in patients with sepsis. In the restrictive arm of the studies, additional fluid administration was permitted only if signs of profound hypoperfusion were detected, in terms of lactate levels > 4 mmol/L, MAP < 50 mmHg, extensive mottling, or urine output < 0.1 mL/kg/h. In both trials, there was no difference detected in the primary outcome of 90-day mortality. Furthermore, a meta-analysis of studies evaluating a restrictive versus a liberal fluid strategy after initial resuscitation in septic patients found that the former was associated with a lower duration of MV, but had no effect on mortality [115]. A previous meta-analysis provided similar results also including acute respiratory distress syndrome (ARDS) and systemic inflammatory response syndrome (SIRS) in addition to sepsis management [116]. Nevertheless, the question remains whether there are specific subgroups of patients who would benefit from a more aggressive or a more conservative IV fluid administration, and if there is a reliable approach to identify them.

Evaluating the heart’s flow response to fluid administration has been proposed to differentiate between fluid responsive and fluid refractory septic states. As static measures, such as CVP, are poor indices of fluid status [24,117], dynamic measures have been employed to predict fluid responsiveness [3,118,119]. Dynamic metrics include passive leg raising (PLR) along with cardiac output (CO) assessment, pulse contour analysis, pulse pressure variation (PPV), stroke volume variation (SVV), and inferior vena cava (IVC) variability with respiration [120]. Passive leg raising to 45° produces hemodynamic changes that mimic volume expansion, and in preload-dependent states, it produces an increase in CO. A >10% increase in CO reliably predicts a fluid-responsive state [121]. In the Fluid Responsiveness Evaluation in Sepsis-associated Hypotension (FRESH) study [122], the researchers compared the use of PLR to guide fluid administration against usual care, and found that the intervention group showed a lower net fluid balance, as well as reduced requirement for renal replacement therapy and MV. The increase in stroke volume (SV) was assessed by a non-invasive bioreactance technology, which has been validated against the more invasive thermodilution method [123,124]. A meta-analysis has confirmed that the PLR challenge detects fluid responsiveness with high sensitivity and specificity [121].

In intubated patients, changes in intrathoracic pressure during MV can be used for the dynamic assessment of fluid responsiveness. Pulse pressure and stroke volume changes between inspiration and expiration, assessed by means of pressure/pulse waveform analysis, have been associated with intravascular volume status and the probability that a volume challenge will increase the SV [125,126]. However, the reliability of these metrics can be limited in certain situations, commonly in the ICU setting, such as the presence of spontaneous breathing, cardiac arrythmias, tidal volume < 8 mL/kg, and reduced lung compliance [127,128]. Doppler echocardiography can also be employed to detect SV changes through aortic velocity time integral measurement, upon bolus fluid administration or PLR manoeuvre, and usually is the most readily available method [129]. Although IVC diameter variation can be easily assessed and has been used to determine preload dependence, reports show that it has limited sensitivity and specificity [130]. Moreover, the standard subcostal measurement of IVC diameter is not always feasible, due to obesity, bowel distention, or presence of surgical wounds. The alternative trans-hepatic approach has been used in these cases, but the results obtained by these two methods are not interchangeable [131]. The measurement of the superior vena cava respiratory variation, although more accurate, it requires the use of transoesophageal Doppler by experienced personnel [132].

Since findings suggest that fluid restriction and negative fluid balance benefits patients with ARDS, whereas liberal fluids are associated with prolonged MV, extravascular lung water (EVLW) measurement has been suggested to guide fluid administration in addition to dynamic measures [128]. EVLW, a marker of pulmonary edema, vascular permeability, and ARDS, is ideally measured by transpulmonary thermodilution, but when not available, lung ultrasonography may provide a rough estimate of lung congestion. In the presence of fluid responsiveness, a fluid challenge will lead to a small increase in EVLW, but in non-responsive states, further fluid administration will result in a large increase in EVLW [13]. A high EVLW during resuscitation informs the physician to maximize efforts towards fluid restriction and to shift to an alternative method for hemodynamic stabilization [133].

Dynamic assessment of fluid responsiveness in a meta-analysis has been associated with a decrease in mortality, ICU length of stay, and duration of MV, but only one study with septic patients was included [134]. In this study, no difference was found in time-to-shock resolution between preload dependence (PLR/PPV) and the control (CVP) arm. A more recent meta-analysis failed to prove a mortality benefit from fluid responsiveness guided treatment in septic patients [52,135]. Consequently, although dynamic assessment has been shown to predict volume responsiveness in sepsis, this has yet to be associated with increased survival, and more studies are needed to assess whether this strategy improves patient-important outcomes.

## 6. Conclusions and Future Directions

Until recently, efforts have been mainly focused on the assessment of macrocircula-tion, such as cardiac output and blood pressure, to guide fluid resuscitation in patients with sepsis, with the exception of CRT. However, the assessment of microcirculation parameters, in relation to microvascular flow and density, tissue perfusion and oxygenation, and glycocalyx integrity has been on the epicenter of intensive research with promising results. Hand-held vital microscopes (HVM) have been investigated for the visualization of the sublingual capillary network, providing a wealth of information in relation to blood flow characteristics, vascular density, and glycocalyx [136,137]. Contrast-enhanced ultrasound (CEUS) is a promising technique for the assessment of the effects of fluid resuscitation and vasopressor administration on the renal microcirculation, as it holds the benefit of directly assessing organ perfusion [138,139]. New methods have also been under research for the assessment of peripheral perfusion, such as laser Doppler flowmetry, a skin blood flow measurement tool [140]. It has been found to correlate well with ICU mortality, and is yet to be tested as a tool to guide resuscitation efforts [141]. These and other methods have been recently reviewed elsewhere [142].

Although fluid management in the septic patient has been extensively studied during the last decades, the “which”, “when”, and “how much” questions are still to be addressed. Evidently, fluid resuscitation has been associated with both benefits and harm; however, there is still a paucity of high-quality evidence to guide the clinical practice regarding fluid management in sepsis. Therefore, controversies remain in the scientific community, and more high-quality, robust studies are warranted to guide future developments. The “one size fits all” approach may be convenient, but it seems that personalized fluid management and taking patient-specific hemodynamic indices into account will provide the basis for better patient outcomes in the future. In this respect, cutting-edge tools for the assessment of perfusion on the tissue level are being investigated and their implementation in real-world settings, however challenging, is expected to revolutionize the management of these patients. Well-designed RCTs are required to compare fluid choices, specifically in septic patients, and they have to be powered enough to evaluate patient subgroups that may respond differently to different types or volume of fluids administered. Existing diagnostic tools have been proven to be valuable in assessing fluid responsiveness, although further trials are yet to determine improvement in patient-important outcomes.

The take-home messages are as follows:

- Data on the optimal type (balanced crystalloids versus normal saline) and volume (liberal versus restricted administration) of fluids in sepsis and septic shock patients are still controversial and elusive.
- Current SSC guidelines recommend the early administration of 30 mL/kg of IV crystalloid fluids for sepsis-related hypotension or a lactate ≥ 4 mmol/L, within the first 3 h of resuscitation. This is a weak recommendation and is based on low-quality evidence.
- Regarding the type of fluid administered during resuscitation, the majority of clinical trials demonstrated no significant difference between the balanced crystalloids and normal saline in the acute kidney injury and mortality.
- Excessive fluid administration during resuscitation can lead to worse outcomes in the septic patient.
- Fluid administration after initial resuscitation should be preferably guided by dynamic measures of fluid responsiveness.
- Fluid management in the critical patient can be optimized by the personalized, bedside, and dynamic assessment of macro- and microcirculation indices.

## Figures and Tables

**Table 1:** Characteristics of the key randomized controlled trials assessing balanced solutions versus normal saline in patients with sepsis and septic shock.

Balanced Solutions versus Normal Saline in Sepsis						
Study ID	Year	Sample Size	Population	Intervention	Comparison	Outcome
**SPLIT** [64]	2015	2262	ICU patients	Plasma-Lyte148 (median volume 2000 mL) N = 1152	NS (median volume 2000 mL) N = 1110	No significant difference in the AKI and mortality within 90 days.
**SALT** [65]	2017	974	ICU adult patients	BS (median volume 1617 mL) N = 520	NS (median volume 1424 mL) N = 454	No significant difference in the AKI and mortality within 30 days. More major kidney events in the NS group.
**SMART** [66]	2019	1641	ICU adult patients	BS Plasma-Lyte A and Lactated Ringer’s (mean volume 2967 mL) N = 824	NS (mean volume 3454 mL) N = 817	Lower incidence of mortality and major adverse kidney events within 30 days in the BS group. Greater number of vasopressor-free days and renal replacement therapy-free days in the BS group.
**BaSICS** [69,70]	2021	10,520	ICU adult patients	BS Plasma-Lyte (median volume 1500 mL) N = 5230	NS (median volume 1500 mL) N = 5290	No significant difference in the AKI and mortality within 90 days. Higher 90-day survival in the subgroup of septic patients receiving balanced crystalloids.
**PLUS** [71]	2022	5037	ICU adult patients	Plasma-Lyte 148 (median volume 3900 mL) N = 2515	NS (median volume 3700 mL) N = 2522	No significant difference in the AKI and mortality within 90 days.
**PRoMPTBOLUS** [72]	Ongoing	Estimated size: 8800	Pediatric patients with sepsis	BS	NS	In progress

AKI: acute kidney injury; BS: balanced solution; ICU: intensive care unit; MV: mechanical ventilation; NS: normal saline.

**Table 2:** Characteristics of the key randomized controlled trials assessing liberal versus restricted fluid administration in patients with sepsis and septic shock.

Liberal versus Restricted Fluid Administration in Sepsis						
Study ID	Year	Sample Size	Population	Intervention	Comparison	Outcome
**EGDT** [40]	2001	263	Adults with sepsis in the ED	Early goal-directed therapy: CVP ≥ 8–12 mmHg, MAP ≥ 65 mmHg, urine ≥ 0.5 mL/kg/h, ScvO_2_ ≥ 70% N = 130	SOC: CVP ≥ 8–12 mmHg, MAP ≥ 65 mmHg, urine ≥ 0.5 mL/kg/h N = 133	Significantly lower in-hospital mortality, APACHE II, SAPS II, and MODS in the EGDT group. Patients in EGDT group received more initial fluids, blood transfusions and inotropic support.
**FEAST** [91]	2011	3141	Children with febrile illness and impaired perfusion	Albumin bolus group N = 1050 Saline bolus group N = 1047	No bolus group N = 1044	Recruitment was halted due to higher 48 h mortality in the intervention arms, and also, higher 4-week mortality in the bolus groups.
**ARISE** [87]	2014	1588	Adults with early septic shock in the ED	EGDT N = 796	SOC N = 792	No difference in 90-day mortality. More patients in the EGDT group received vasopressors, but no other significant differences were observed.
**ProCESS** [88]	2014	902	Adults in the ED with SIRS and refractory hypotension or hyperlactemia	EGDT N = 456	SOC N = 446	No difference in 60-day, 90-day, 1-year mortality, or need for organ support
**ProMISe** [89]	2015	1260	Adults >6 h in the ED with infection, refractory hypotension or hyperlactemia	EGDT N = 630	SOC N = 630	No difference in 90-daysmortality. Significantly higher cardiovascular support and length of ICU stay in the EGDT group.
**SSSP-2** [93]	2017	212	Adults in ED with suspected sepsis and hypotension	Fluid administration guided by SpO_2_, RR, and JVP (total up to 4 L) N = 107	Usual care N = 105	Intervention arm received more fluids and vasopressors. Higher in-hospital, 28-day mortality and worsening hypoxemia in the intervention group.
**FRESH** [104]	2020	124	Adults with sepsis-associated hypotension in ED	Assessment of fluid responsiveness before fluid administration PLR test, SV change ≥ 10% N = 83	Usual care N = 41	Similar volume of resuscitation fluids and ICU length of stay in the two arms. Significantly less positive fluid balance, RRT, and MV in the intervention group.
**CLASSIC** [95]	2022	1554	Adults with septic shock in ICU	Restrictive fluid group Fluids guided by lac, MAP, urine output, mottling, losses, dehydration, and electrolyte disturbances N = 770	Liberal fluids according to SOC N = 784	No difference in 90-day mortality or serious adverse events between the two group.
**CLOVERS** [96]	2023	1563	Adults with infection and refractory hypotension	Restrictive fluid group Vasopressor prioritization Only “rescue fluids” for prespecified indications N = 782	Liberal fluids according to SOC N = 781	No difference in mortality before discharge home by day 90 between the two arms. Similar frequency of adverse events.

APACHE: Acute Physiology and Chronic Health Evaluation; CVP: central venous pressure; ED: emergency department; EGDT: early goal-directed therapy; ICU: intensive care unit; JVP: jugular venous pressure; MAP: mean arterial pressure; MODS: Multiple Organ Dysfunction Score; MV: mechanical ventilation; PLR: passive leg raising; RR: respiratory rate; RRT: renal replacement therapy; SAPS: Simplified Acute Physiology Score; ScvO_2_: central venous oxygen saturation; SIRS: systemic inflammatory response syndrome; SOC: standard of care; SpO_2_: oxygen saturation; SV: stroke volume.

## Data Availability

The data underlying this article will be shared on reasonable request to the corresponding author.

## Abbreviations

AKI	Acute kidney injury
ALBIOS	Albumin Italian Outcome Sepsis
APACHE	Acute Physiology and Chronic Health Evaluation
ARDS	Acute respiratory distress syndrome
BaSICS	Balanced Solution in Intensive Care Study
BS	Balanced Solution
CEUS	Contrast-enhanced ultrasound
CLASSIC	Conservative versus Liberal Approach to Fluid Therapy of Septic Shock in Intensive Care
CLOVERS	Crystalloid Liberal or Vasopressors Early Resuscitation in Sepsis
CO	Cardiac output
CRT	Capillary refill time
CVP	Central venous pressure
DAMPs	Danger-associated molecular patterns
ED	Emergency department
EGDT	Early goal-directed therapy
ESICM	European Society of Intensive Care Medicine
EVLW	Extravascular lung water
FEAST	Fluid Expansion as Supportive Therapy
FRESH	Fluid Responsiveness Evaluation in Sepsis-associated Hypotension
ICU	Intensive care unit
HES	Hydroxyethyl starch
HIV	Human immunodeficiency virus
HVM	Hand-held vital microscopes
IL-1β	Interleukin-1 beta
IL-6	Interleukin-6
IV	Intravenous
IVC	Inferior vena cava
JVP	Jugular venous pressure
MAP	Mean arterial pressure
MODS	Multiple Organ Dysfunction Score
MV	Mechanical ventilation
NS	Normal saline
PAMPs	Pathogen-associated molecular patterns
PLR	Passive leg raising
PLUS	Plasma-Lyte 148 versus Saline Study
PPV	Pulse pressure variation
ProCESS	Protocolized Care for Early Septic Shock
ProMISe	Protocolised Management in Sepsis
PRoMPT BOLUS	Pragmatic Pediatric trial of Balanced versus NS Fluid in sepsis
PRRs	Pattern recognition receptors
RCTs	Randomized controlled trials
ROSE	Resuscitation, optimization, stabilization, and evacuation
RR	Respiratory rate
RRT	Renal replacement therapy
SAFE	Saline versus Albumin Fluid Evaluation
SALT	Isotonic Solution Administration Logistical Testing
SAPS	Simplified Acute Physiology Score
ScvO2	Central venous oxygen saturation
SIRS	Systemic inflammatory response syndrome
SMART	Isotonic Solutions and Major Adverse Renal Events Trial
SOC	Standard of care
SOFA	Sequential organ failure assessment
SPLIT	Saline vs. Plasma-Lyte 148 for ICU fluid Therapy
SpO2	Oxygen saturation
SSC	Surviving Sepsis Campaign
SSSP	Simplified Severe Sepsis Protocol
SSSP-2	Simplified Severe Sepsis Protocol 2
SV	Stroke volume
SVR	Systemic vascular resistance
SVV	Stroke volume variation
TLRs	Toll-like receptors
TNF-a	Tumor necrosis factor alpha
